# Supplementary material for: Retention and Molecular Evolution of Lipoxygenase Genes in Modern Rosid Plants
Source: Front Genet. 2016 Sep 30;7:176. doi: 10.3389/fgene.2016.00176 (PMC5043136; doi:10.3389/fgene.2016.00176)
Supplement: Supplementary file 1 [file DataSheet1.PDF]

Supplementary Material

Retention and molecular evolution of lipoxygenase genes in modern rosid plants

Zu Chen[*], Danmei Chen, Wenyuan Chu, Dongyue Zhu, Hanwei Yan, Yan Xiang

*** Correspondence:** Yan Xiang: xiangyanahau@sina.com

Supplementary Figure 1

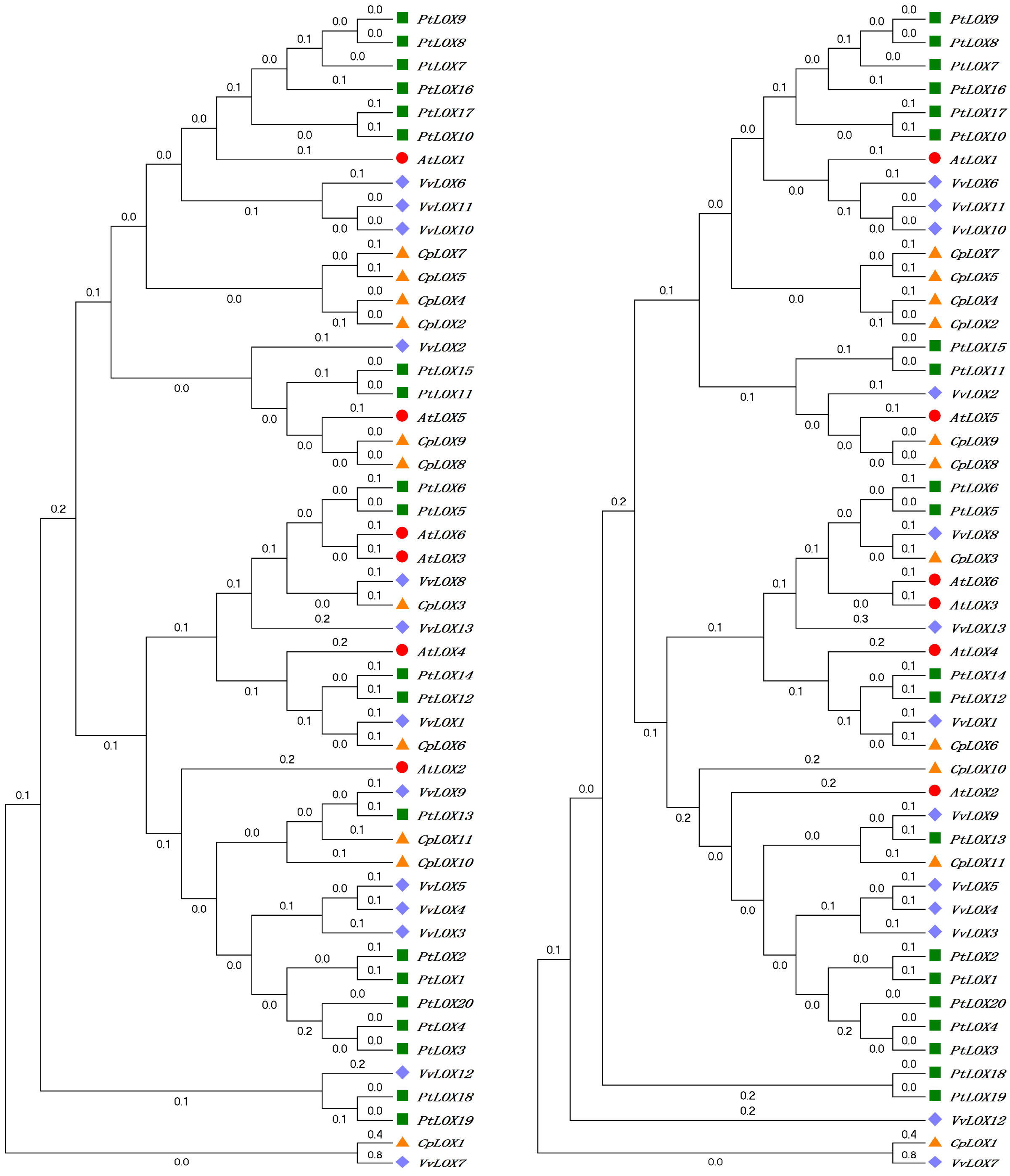

Supplementary Figure 1. Phylogenetic tree of LOXs constructed by two methods with MEGA for four modern rosids. The left phylogenetic tree of LOX constructed by the ME method and the right one constructed by NJ methods.

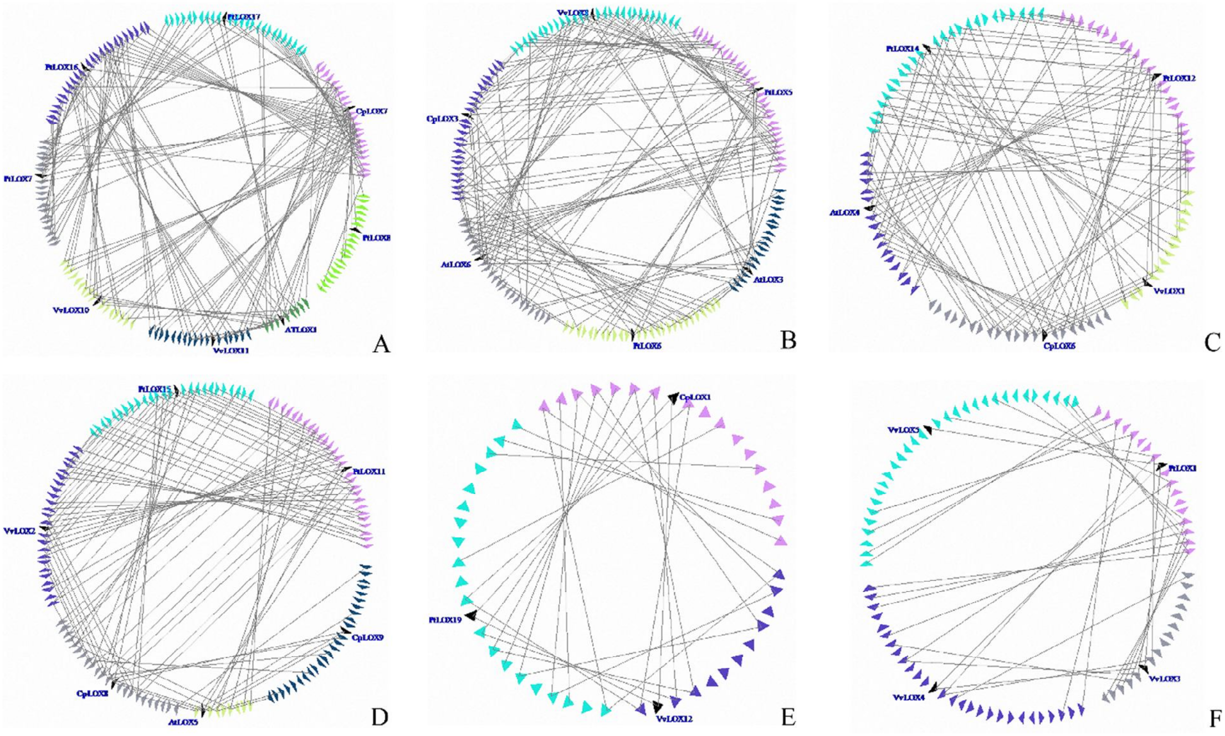

Supplementary Figure 2. The full syntenic maps of chromosome regions containing LOX genes across four rosid plants.

Supplementary Table 1. Identity values between the gene models and EST sequences of CpLOXs.

Gene name	mRNA sequence ID	Identity
CpLOX1	gi|186734020|gb|EX251804.1|EX251804	100
CpLOX2	gi|186850026|gb|EX302886.1|EX302886	98.43
CpLOX3	gi|186745685|gb|EX277262.1|EX277262	99.6
CpLOX4	gi|186825465|gb|EX262116.1|EX262116	99.14
CpLOX5	gi|186802236|gb|EX257681.1|EX257681	99.76
CpLOX6	gi|186854896|gb|EX265398.1|EX265398	99.03
CpLOX7	gi|186799692|gb|EX291269.1|EX291269	98.87
CpLOX8	gi|186784733|gb|EX230759.1|EX230759	100
CpLOX9	gi|186784733|gb|EX230759.1|EX230759	93.11
CpLOX10	gi|186711487|gb|EX253823.1|EX253823	100
CpLOX11	gi|186851425|gb|EX258978.1|EX258978	99.35

Supplementary Table 2. Plant lipoxygenase sequences used for multiple sequence alignment with gene models of CpLOXs

Organism	Sequence ID	GeneBank Accession
Arabidopsis thaliana	*AtLOX1*	Q06327
	AtLOX2	P38418
	AtLOX3	Q9SMW1
	AtLOX4	Q9FNX8
	AtLOX5	Q9FNX7
	AtLOX6	Q9CAG3
Cucumis sativus	*CsLOX2*	AAA79186
	CsLOX3	CAA63483
	CsLOX4	CAB83038
Glycine max	*GmLOX1*	P08170
	GmLOX2	P09435
	GmLOX3	P09186
	GmLOX4	P38417
	GmLOX6	AAA96817
	GmLOX7	P24095
Hordeum vulgare	*HvLOX1*	P93184
	HvLOX2	Q8GSM3
	HvLOX3	Q8GSM2
	HvLOX4	CAI84707
	HvLOXA	P29114
	HvLOXB	AAB60715
	HvLOXC	AAB70865
Lens culinaris	*LcLOX1*	P38414
Lycopersicon esculentum	*LeLOX1*	P38415
	LeLOX2	P38416
	LeLOX3	AAG21691

	LeLOX4	Q96573
	LeLOX5	Q96574
Nicotiana attenuata	*NaLOX1*	AAP83136
	NaLOX2	AAP83137
	NaLOX3	AAP83138
Nicotiana tabacum	*NtLOX1*	CAA58859
Oryza sativa	*OsLOX1*	Q76I22
	OsLOX10	Q0DJB6
	OsLOX2	P29250
	OsLOX2.3	Q6H7Q6
	OsLOX3	Q7G794
	OsLOX3b	Q53RB0
	OsLOX5	Q7XV13
	OsLOX6	Q8H016
	OsLOX7	P38419
	OsLOX8	Q84YK8
	OsLOX9	Q01S17
	OsLOXRCI1	Q9FSE5
Pisum sativum	*PsLOX1*	AAB71759
	PsLOX2	P14856
	PsLOX3	P09918
	PsLOX7	CAC04380
	PsLOX8	CAA75609
	PsLOX9	CAG44504
	PsLOXG	CAA53730
Phaseolus vulgaris	*PvLOX1*	P27480
	PvLOX1b	AAB18970
	PvLOX2b	AAG42354
	PvLOX2c	AAF15396
Solanum tuberosum	*StLOX1*	CAA64765
	StLOX2	AAD09202
	StLOX3	AAB67865
	StLOX4	CAA65268
	StLOX5	CAA65269
Zea mays	*ZmLOX1*	AAL73499
	ZmLOX2	AAF76207
Triticum aestivum L.	*TaLOX1*	GQ166692
	TaLOX2	GQ166691
Actinidia deliciosa	*AdLOX1*	DQ497792
	AdLOX2	DQ497797
	AdLOX3	DQ497795
	AdLOX4	DQ497793
	AdLOX5	DQ497796
	AdLOX6	DQ497794

Supplementary Table 3. Identity values between the gene models of CpLOXs and experimantally verified gene models.

Gene name	clone sequence ID	Identity
CpLOX1	*OsLOX10*	42.57
CpLOX2	*NaLOX1*	62.21
CpLOX3	*NaLOX3*	74.44
CpLOX4	*StLOX3*	64.7
CpLOX5	*NtLOX1*	67.14
CpLOX6	*AtLOX4*	60.69
CpLOX7	*StLOX3*	66.04
CpLOX8	*AtLOX5*	68.65
CpLOX9	*AtLOX5*	70.29
CpLOX10	*NaLOX2*	61.58
CpLOX11	*NaLOX2*	65.69

Supplementary Table 4. Detailed signature matches of LOX gene in Carica Papaya

Gene Name	Protein family membership	Detailed signature matches						
	Interpro	SMART	PROSITE	CATH	pFAM	Superfamily	GO	SPRINT
CpLOX1	LOX, plant (IPR001246)	SM00308 (LH2_4)	PS51393 (LOX) PS50095 (PLAT)	G3DSA:2.60.60.20 (PLAT)	PF00305 (LOX) PF01477 (PLAT)	SSF48484 (LOX) SSF49723 (PLAT/LH2 domain)	GO:0055114 oxidation reduction process	PR00087 (LOX)
CpLOX2	LOX, plant (IPR001246)	SM00308 (LH2_4)	PS51393 (LOX) PS50095 (PLAT)	G3DSA:2.60.60.20 (PLAT)	PF00305 (LOX) PF01477 (PLAT)	SSF48484 (LOX) SSF49723 (PLAT/LH2 domain)	GO:0055114 oxidation reduction process	PR00087 (LOX)
CpLOX3	LOX, plant (IPR001246)	SM00308 (LH2_4)	PS51393 (LOX) PS50095 (PLAT)	G3DSA:2.60.60.20 (PLAT)	PF00305 (LOX) PF01477 (PLAT)	SSF48484 (LOX) SSF49723 (PLAT/LH2 domain)	GO:0055114 oxidation reduction process	PR00087 (LOX)
CpLOX4	LOX, plant (IPR001246)	SM00308 (LH2_4)	PS51393 (LOX) PS50095 (PLAT)	G3DSA:2.60.60.20 (PLAT)	PF00305 (LOX) PF01477 (PLAT)	SSF48484 (LOX) SSF49723 (PLAT/LH2 domain)	GO:0055114 oxidation reduction process	PR00087 (LOX)
CpLOX5	LOX, plant (IPR001246)	SM00308 (LH2_4)	PS51393 (LOX) PS50095 (PLAT)	G3DSA:2.60.60.20 (PLAT)	PF00305 (LOX) PF01477 (PLAT)	SSF48484 (LOX) SSF49723 (PLAT/LH2 domain)	GO:0055114 oxidation reduction process	PR00087 (LOX)
CpLOX6	LOX, plant (IPR001246)	SM00308 (LH2_4)	PS51393 (LOX) PS50095 (PLAT)	G3DSA:2.60.60.20 (PLAT)	PF00305 (LOX) PF01477 (PLAT)	SSF48484 (LOX) SSF49723 (PLAT/LH2 domain)	GO:0055114 oxidation reduction process	PR00087 (LOX)
CpLOX7	LOX, plant (IPR001246)	SM00308 (LH2_4)	PS51393 (LOX) PS50095 (PLAT)	G3DSA:2.60.60.20 (PLAT)	PF00305 (LOX) PF01477 (PLAT)	SSF48484 (LOX) SSF49723 (PLAT/LH2	GO:0055114 oxidation reduction process	PR00087 (LOX)

						domain)		
CpLOX8	LOX, plant (IPR001246)	SM00308 (LH2_4)	PS51393 (LOX) PS50095 (PLAT)	G3DSA:2.60.60.20 (PLAT)	PF00305 (LOX) PF01477 (PLAT)	SSF48484 (LOX) SSF49723 (PLAT/LH2 domain)	GO:0055114 oxidation reduction process	PR00087 (LOX)
CpLOX9	LOX, plant (IPR001246)	SM00308 (LH2_4)	PS51393 (LOX) PS50095 (PLAT)	G3DSA:2.60.60.20 (PLAT)	PF00305 (LOX) PF01477 (PLAT)	SSF48484 (LOX) SSF49723 (PLAT/LH2 domain)	GO:0055114 oxidation reduction process	PR00087 (LOX)
CpLOX10	LOX, plant (IPR001246)	SM00308 (LH2_4)	PS51393 (LOX) PS50095 (PLAT)	G3DSA:2.60.60.20 (PLAT)	PF00305 (LOX) PF01477 (PLAT)	SSF48484 (LOX) SSF49723 (PLAT/LH2 domain)	GO:0055114 oxidation reduction process	PR00087 (LOX)
CpLOX11	LOX, plant (IPR001246)	SM00308 (LH2_4)	PS51393 (LOX) PS50095 (PLAT)	G3DSA:2.60.60.20 (PLAT)	PF00305 (LOX) PF01477 (PLAT)	SSF48484 (LOX) SSF49723 (PLAT/LH2 domain)	GO:0055114 oxidation reduction process	PR00087 (LOX)

Supplementary Table 5. Ks values for the paralogous and orthologous pairs of LOXs.

	Paralogous pair	Ka	Ks	Ka/Ks
Arabidopsis thaliana	*AtLOX3-AtLOX6*	0.10	0.98	0.10
Carica papaya	*CpLOX2-CpLOX4*	0.06	0.36	0.17
	CpLOX2-CpLOX5	0.14	0.88	0.16
	CpLOX2-CpLOX7	0.14	0.84	0.17
	CpLOX4-CpLOX5	0.15	0.96	0.15
	CpLOX4-CpLOX7	0.14	0.89	0.16
	CpLOX5-CpLOX7	0.13	0.71	0.18
	CpLOX8-CpLOX9	0.04	0.17	0.27
	CpLOX10-CpLOX11	0.21	0.95	0.22
Vitis vinifera	*VvLOX3-VvLOX4*	0.17	0.33	0.52
	VvLOX3-VvLOX5	0.23	0.53	0.43
	VvLOX4-VvLOX5	0.16	0.59	0.28
	VvLOX4-VvLOX9	0.25	1.45	0.17
	VvLOX6-VvLOX10	0.06	0.08	0.83
	VvLOX10-VvLOX11	0.01	0.06	0.27
	VvLOX10-VvLOX2	0.29	1.17	0.25
	VvLOX10-VvLOX6	0.06	0.08	0.83
	VvLOX11-VvLOX2	0.25	1.04	0.24
	VvLOX11-VvLOX6	0.08	0.07	1.16
	VvLOX12-VvLOX7	0.17	0.18	0.96
Populus trichocarpa	*PtLOX1-PtLOX2*	0.14	0.53	0.27
	PtLOX3-PtLOX4	0.00	0.01	0.20
	PtLOX5-PtLOX6	0.07	0.03	0.25
	PtLOX7-PtLOX10	0.10	0.33	0.31
	PtLOX7-PtLOX16	0.05	0.26	0.21
	PtLOX7-PtLOX8	0.02	0.04	0.43
	PtLOX7-PtLOX9	0.01	0.02	0.38
	PtLOX8-PtLOX10	0.11	0.30	0.36
	PtLOX8-PtLOX16	0.06	0.27	0.24
	PtLOX8-PtLOX9	0.02	0.05	0.42
	PtLOX10-PtLOX16	0.10	0.37	0.28
	PtLOX10-PtLOX17	0.12	0.29	0.41
	PtLOX10-PtLOX9	0.11	0.34	0.31
	PtLOX11-PtLOX15	0.04	0.21	0.20
	PtLOX11-PtLOX7	0.20	1.48	0.13
	PtLOX11-PtLOX9	0.20	1.45	0.14
	PtLOX12-PtLOX14	0.06	0.25	0.22
	PtLOX15-PtLOX7	0.20	1.49	0.13
	PtLOX15-PtLOX9	0.20	1.45	0.14

PtLOX16-PtLOX9	0.05	0.26	0.21
PtLOX17-PtLOX16	0.10	0.34	0.31
PtLOX18-PtLOX19	0.02	0.04	0.52
PtLOX20-PtLOX3	0.01	0.04	0.26
PtLOX20-PtLOX4	0.01	0.04	0.33

Orthologous pair	Ka	Ks	Ka/Ks
PtLOX12-AtLOX4	0.25	1.68	0.15
PtLOX14-AtLOX4	0.26	1.59	0.16
VvLOX1-AtLOX4	0.26	1.92	0.14
VvLOX10-PtLOX11	0.18	1.10	0.16
VvLOX10-PtLOX15	0.17	0.99	0.18
VvLOX10-PtLOX16	0.14	0.95	0.15
VvLOX10-PtLOX9	0.13	0.95	0.14
VvLOX11-PtLOX16	0.14	1.10	0.13
VvLOX11-PtLOX7	0.12	1.11	0.11
VvLOX11-PtLOX9	0.12	1.11	0.11
VvLOX12-PtLOX18	0.22	0.68	0.32
VvLOX12-PtLOX19	0.22	0.70	0.31
VvLOX1-PtLOX12	0.14	0.93	0.16
VvLOX1-PtLOX14	0.15	0.96	0.16
VvLOX2-PtLOX11	0.19	0.72	0.26
VvLOX2-PtLOX15	0.19	0.73	0.26
VvLOX2-PtLOX7	0.24	1.17	0.21
VvLOX2-PtLOX9	0.24	1.12	0.22
VvLOX4-PtLOX1	0.28	1.29	0.22
VvLOX4-PtLOX13	0.25	1.98	0.12
VvLOX4-PtLOX2	0.24	1.14	0.21
VvLOX5-PtLOX2	0.23	1.10	0.21
VvLOX6-PtLOX10	0.27	0.43	0.62
VvLOX6-PtLOX7	0.22	0.43	0.52
VvLOX6-PtLOX8	0.22	0.47	0.47
VvLOX6-PtLOX9	0.22	0.44	0.50
VvLOX8-PtLOX5	0.15	1.65	0.09
VvLOX8-PtLOX6	0.18	1.72	0.10
VvLOX9-PtLOX13	0.19	1.07	0.18

Supplementary Table 6. Detailed information about the 20 motifs

MOTIF	WIDTH	BEST POSSIBLE MATCH	E-Value
1	93	WQLAKAHVCVNDSGYHQLVSHWLRTHCCMEPFIIATNRQLSVMHPIYK LLHPHFRYTMNINALARQILINAGGIIESCFFPGKYCMEMSSAAY	9.3E-60
2	73	ELQAWWTEVRNVGHGDKKDEPWWPKMQTCEDLIQICTTIIWVASAHHAAVNFG QYPYAGYFPNRPTIMRRFMP	1.4E-43
3	41	DPTAPHGVRLLIEDYPYANDGLEIWSAIKTWVRDYCNHYYP	3E-20
4	70	GPVHFVCNSWVYPKKDNPKKRIFFTNKSYLPSQTPAGLRKYREEELMNL RGNGKGERKEWDRVYDYDYYN	0.00021/7.1E-12
5	28	DKFAWRRDEEFARQMLAGVNPVVIQRLQ	2.1E-10
6	29	TKTYASRTLFFLTDDGTLKPLAIELSLPH	8.2E-11
7	38	FEKFSAKLQEIEKIIDERNADKKLKNRCGAGVVPYELL	2.5E-12
8	47	GDPEYAEFLSNPQKYFLKCIPSQLQATKGMAVIDILSRHSPDEEYLG	7.5E-15
9	41	EVAYKVTFDWDEDFGEPGAFLVCNNHHSEFYLKTITLEGFP	0.00001
10	41	MSLNIYVPRDERFSHVKMSTFSAYALKSVVHFLIPEIEALC	3.4E-09
11	29	MTVDQAMEQNRLFILDHHDMLMPYLRRIN	3.7E-11
12	21	MWRFDMQALPADLIKRGMAVE	0.00000011
13	15	GSKEYPYPRRCRTGR	
14	29	EWPPKSKLDPEIYGPQNSAITKEHIEQNM	0.000000035
15	46	NEFDSFQDVMDLYEGGIKLPNGPLLDNIRDNIPWEMLKEIFRTDGE	0.00013
16	15	EPGVTGRGIPNSISI	
17	34	RIFFTNQSYLPSETPEGLRKYREEELVNLRGNGK	
18	29	KIKGTVVLMKKNVLDFNDFNASVLDRVHE	
19	29	KEWDRIYDYDYYNDLGNPDKGPEYARPVL	2.8E-11
20	29	DPANGLQGKLGKPAYLENWITTITSLTAG	E-Value
